# Nonsense Mediated Decay Resistant Mutations Are a Source of Expressed Mutant Proteins in Colon Cancer Cell Lines with Microsatellite Instability

**DOI:** 10.1371/journal.pone.0016012

**Published:** 2010-12-31

**Authors:** David S. Williams, Matthew J. Bird, Robert N. Jorissen, Yen Lin Yu, Franscesa Walker, Hui Hua Zhang, Edouard C. Nice, Antony W. Burgess

**Affiliations:** 1 Epithelial Biochemistry Laboratory, Ludwig Institute for Cancer Research, Melbourne Branch, Parkville, Victoria, Australia; 2 Department of Pathology, University of Melbourne, Parkville, Victoria, Australia; 3 Department of Anatomical Pathology, Melbourne Health, Parkville, Victoria, Australia; 4 Ludwig Colon Cancer Initiative Laboratory, Ludwig Institute for Cancer Research, Melbourne Branch, Parkville, Victoria, Australia; Chinese University of Hong Kong, Hong Kong

## Abstract

**Background:**

Frameshift mutations in microsatellite instability high (MSI-High) colorectal cancers are a potential source of targetable neo-antigens. Many nonsense transcripts are subject to rapid degradation due to nonsense-mediated decay (NMD), but nonsense transcripts with a cMS in the last exon or near the last exon-exon junction have intrinsic resistance to nonsense-mediated decay (NMD). NMD-resistant transcripts are therefore a likely source of expressed mutant proteins in MSI-High tumours.

**Methods:**

Using antibodies to the conserved N-termini of predicted mutant proteins, we analysed MSI-High colorectal cancer cell lines for examples of naturally expressed mutant proteins arising from frameshift mutations in coding microsatellites (cMS) by immunoprecipitation and Western Blot experiments. Detected mutant protein bands from NMD-resistant transcripts were further validated by gene-specific short-interfering RNA (siRNA) knockdown. A genome-wide search was performed to identify cMS-containing genes likely to generate NMD-resistant transcripts that could encode for antigenic expressed mutant proteins in MSI-High colon cancers. These genes were screened for cMS mutations in the MSI-High colon cancer cell lines.

**Results:**

Mutant protein bands of expected molecular weight were detected in mutated MSI-High cell lines for NMD-resistant transcripts (CREBBP, EP300, TTK), but not NMD-sensitive transcripts (BAX, CASP5, MSH3). Expression of the mutant CREBBP and EP300 proteins was confirmed by siRNA knockdown. Five cMS-bearing genes identified from the genome-wide search and without existing mutation data (SFRS12IP1, MED8, ASXL1, FBXL3 and RGS12) were found to be mutated in at least 5 of 11 (45%) of the MSI-High cell lines tested.

**Conclusion:**

NMD-resistant transcripts can give rise to expressed mutant proteins in MSI-High colon cancer cells. If commonly expressed in primary MSI-High colon cancers, MSI-derived mutant proteins could be useful as cancer specific immunological targets in a vaccine targeting MSI-High colonic tumours.

## Introduction

Approximately 15% of colorectal, gastric and endometrial cancers have defective DNA mismatch repair and high-level microsatellite instability (MSI-High)[1], [2]. Most MSI-High tumours result from sporadic hypermethylation of the MLH1 gene promoter[3], but mutation of a DNA mismatch repair enzyme is the underlying defect in cases of hereditary non-polyposis colorectal cancer (HNPCC)[4]. Tumours with MSI have a form of genetic instability that manifests as frameshift mutations in repetitive microsatellite sequences of DNA[1]. Frameshift mutations in coding microsatellites (cMS) alter the genetic reading frame and in most instances encode for truncated proteins with unique C-terminal protein sequences.

An intriguing pathological feature of MSI-High colorectal cancers is a tendency to have increased numbers of tumour infiltrating lymphocytes (TILs) relative to MSI-Low and microsatellite stable (MSS) tumours[5]. The TILs in MSI-High tumours are activated CD8+ cytotoxic T lymphocytes (CTLs)[6], suited to recognising intracellular peptides such as MSI-derived neo-antigens, presented by HLA class I molecules. An MSI-High phenotype and increased TILs in a colorectal cancer connote a better stage-matched prognosis relative to MSI-Low or MSS tumours[7], [8]. This survival advantage may reflect a protective benefit of the immune infiltrate[9].

Synthetic peptides corresponding to mutant proteins predicted to arise from MSI-associated frameshift mutations have been shown to be immunogenic. Over the past decade, cytotoxic T lymphocyte (CTL) responses have been described against HLA-A2 binding peptides arising from frameshift mutations in the A10 cMS of TGFBR2[10], [11], the A10 cMS of CASP5[12], the T10 cMS of OGT[13] and recently the T15 cMS of U79260(FTO)[14]. CTL responses to mutant proteins have been detected in patients with MSI associated colon cancers and in healthy HNPCC-mutation carriers, raising the possibility of protective immunosurveillance in the latter population[15].

There is published mutation data available for at least 1522 mononucleotide coding microsatellites from 460 genes in MSI-High colorectal cancers[16]. A correlation exists between cMS length and mutation rate. Genes with cMS mutations are potential sources of targetable proteins for immune therapy strategies. Since frameshift mutations affect cMS repeats in a predictable manner, the mutant proteins generated are likely to be conserved in a population of patients with MSI-High colon cancers[17], [18]. Although no one mutant transcript is common to all MSI-High tumours, a vaccine that targets a set of commonly mutated proteins may be an effective treatment for MSI-High colon cancers. There is a need for new therapies against MSI-High colon cancers, since multiple studies have shown these tumours to be resistant to standard chemotherapeutics such as 5-fluorouracil[19], [20], [21].

The existence of MSI-derived mutant proteins has been inferred by immunological studies, but there is a lack of published data regarding the direct detection of the predicted MSI-derived mutant proteins. Western blot analyses have been published in which potential mutant protein bands were detected for the CREBBP, EP300 [22] and MBD4 genes [23], but there was no validation of these bands. Confirming the expression of mutant proteins in MSI-High tumours is important if these are to be successful therapeutic targets for a vaccine. Stable expression of mutant proteins which facilitates cross presentation of tumour antigens by professional antigen presenting cells and stimulates a T helper response is likely to provide superior anti-tumour immunity[18]. However, most mutant transcripts generated by MSI are targeted for rapid degradation by nonsense-mediated decay (NMD)[24], [25] and in the absence of intervention, little to no targetable mutant protein will be generated from these aberrant transcripts[24].

In this study we have detected three MSI-derived mutant proteins, all of which arise from NMD-resistant transcripts. Following a genome-wide search for additional NMD-resistant transcripts, cMS-containing genes were screened for mutations in a panel of 11 MSI-High colon cancer cell lines. Genes were selected for mutation analysis on the basis of likely high mutation frequency (based on cMS length), likely immunogenicity (based on mutant C-terminus amino acid length) and likely expression in colon cancer (based on gene and protein expression data where available). Commonly mutated NMD-resistant transcripts were assessed for probable HLA-A*0201 CTL epitopes using *in silico* predictive software, SYFPEITHI[26], for comparison with MSI-derived epitopes already shown to be immunogenic *in vitro*.

Our results confirm that NMD-resistant transcripts give rise to expressed mutant proteins and also show that commonly occurring frameshift mutations that generate NMD-resistant transcripts are a promising source of targetable tumour antigens in MSI-High colon cancers. A set of commonly expressed mutant proteins has the potential to form the basis for a multivalent vaccine or immunotherapeutic targeting MSI-High tumours.

## Results

### MSI-High colon cancer cell lines harbor frameshift mutations in multiple cMS

Screening of the colon cancer cells lines for aberrant expression DNA mismatch repair proteins revealed an abnormal phenotype consistent with MSI in 11 of 17 cell lines (Table 1). The cell lines were assessed for frameshift mutations in a selection of genes with cMS previously reported to develop mutations in MSI-High colon cancers. Numerous mutations consistent with an MSI-High phenotype were detected in each cell line with aberrant DNA mismatch repair expression by immunohistochemistry, but few mutations if any were detected in the remaining cell lines (Table 2, Table S1). Deletion of 1 DNA base pair was the most common mutation, accounting for 82% (214/262) of the mutations observed in this study (probable biallelic mutations counted as 2 mutations). Most frameshift mutations encode for truncated proteins of lower molecular weight with a mutated C-terminus (Table 3).

**Table 1:** Assessment of cell lines for MSI status by IHC.

Cell line	MSI status	MLH1	PMS2	MSH2	MSH6	Reference
SW1222	MSS	normal	-	normal	normal	[52]
SW480	MSS	normal	-	normal	normal	[52]
LIM1863	MSS	normal	normal	normal	normal	[47]
LIM2099	MSS	normal	normal	normal	normal	[49]
LIM2463	MSS	normal	normal	normal	normal	[48]
HT29	MSS	normal	-	normal	Normal	[53]
HCA-7	MSI	absent	-	normal	normal	[54]
HCT116	MSI	absent	-	normal	normal	[55]
LoVo	MSI	normal	-	absent	absent	[56]
LS174T	MSI	absent	-	normal	normal	[57]
LIM1215	MSI	absent	-	normal	normal	[45]
LIM1899	MSI	normal	absent	normal	normal	[46]
LIM2405	MSI	absent	absent	normal	normal	[49]
LIM2408	MSI	absent	absent	normal	normal	[49]
LIM2537	MSI	absent	absent	normal	normal	[49]
LIM2550	MSI	absent	absent	normal	normal	[49]
LIM2551	MSI	absent	absent	normal	normal	[49]

**Table 2:** Representative cMS mutation analysis in colon cancer cell lines.

Gene	BAX	MSH3	TGFBR2	CASP5	MBD4	ACVR2A	ACVR2A	CREBBP	EP300	AIM2	TTK	PTHLH	TCF7L2
cMS	G8	A8	A10	A10	A10	A8 (1st)	A8 (2nd)	C5	A5	A10	A9	A11	A9
Cell line	NMD-S	NMD-S	NMD-S	NMD-S	NMD-S	NMD-S	NMD-R	NMD-R	NMD-R	NMD-R	NMD-R	NMD-R	NMD-R
SW1222	wt	wt	wt	wt	wt	wt	wt	wt	wt	wt	wt	-	wt
SW480	wt	wt	wt	wt	wt	wt	wt	wt	wt	wt	wt	wt	wt
LIM1863	wt	wt	wt	wt	wt	wt	−1	wt	wt	wt	wt	wt	wt
LIM2099	wt	wt	wt	wt	wt	wt	wt	wt	wt	wt	wt	wt	wt
LIM2463	wt	wt	wt	wt	wt	wt	−1, wt	wt	wt	wt	wt	wt	wt
HT29	wt	wt	wt	wt	wt	wt	wt	wt	wt	wt	wt	wt	wt
HCA-7	−1	−1	−1	+1, wt	−1	wt	−1	wt	wt	−1, wt	wt	−2, wt	+1, wt
HCT116	−1	−1	−1	−1, wt	−1, wt	wt	−1	wt	−1, wt	−1, wt	−1, wt	−2	wt
LoVo	−1, +1	wt	−2, −1	−1, wt	wt	wt	−1	−1, wt	wt	−1	−1, wt	−1	−1, wt
LS174T	−1	wt	−2, −1	−1	wt	wt	−1	wt	wt	wt	−1, wt	−1, wt	−1, wt
LIM1215	−1	−1, wt	−1	−1	wt	wt	−1, wt	wt	wt	−1, wt	−1, wt	−1	wt
LIM1899	−1	wt	−2, +1	−1, wt	−1, wt	wt	−1	wt	wt	wt	−1, wt	−1, wt	wt
LIM2405	wt	−1, wt	wt	−2, −1	wt	−1	−1, wt	wt	wt	wt	wt	−1, wt	wt
LIM2408	wt	−1, wt	wt	−2, −1	wt	−1	−1, wt	wt	wt	−1, wt	wt	−1, wt	−1, wt
LIM2537	−1, wt	wt	−2, −1	−2, wt	−1, wt	wt	−1	wt	wt	−2, −1	−1, wt	−1, wt	wt
LIM2550	wt	wt	−1, wt	−1, wt	−1, wt	wt	−1	wt	wt	−2, −1	−1, wt	−1, wt	wt
LIM2551	wt	wt	−1	−2, wt	wt	wt	−1, wt	wt	wt	−1, wt	−1, wt	−1, wt	−1, wt

−1 = deletion of 1 base in cMS; wt = wild type. NMD-S: predicted NMD-sensitive mutant transcript; NMD-R: predicted NMD-resistant mutant transcript. ACVR2A: A8 (1^st^) and A8 (2^nd^) refer to two separate cMS in order of coding sequence.

**Table 3:** Mutant C-terminal peptide sequences encoded for by frameshift mutations in (A) NMD-sensitive and (B) NMD-resistant transcripts.

A.					
Gene	cMS	MW(w.t.)	MW(mut)	a.a.	Mutant neo C-terminus (−1 DNA base pair frameshift)
TAF1B	A11	68143	9547	25	TILKKAGIGMCVKVSSIFFINKQKP
TGFBR2	A10	64568	17931	34	SLVRLSSCVPVALMSAMTTSSSQKNITPAILTCC
CASP5	A10	49736	10813	25	QLRCWNTWAKMFFMVFLIIWQNTMF
MSH3	A8	128029	45890	31	RATFLLALWECSLPQARLCLIVSRTLLLVQS
BAX	G8	21184	6394	18	RHPSWPWTRCLRMRPPRS*
MBD4	A10	66050	34937	3	KDH
IGF2R	G8	274374	147290	26	TLAIRFISAPQPSSSTVTAAPSGQYF
ACVR2A	A8(1)	57847	16870	53	TALKYIFVAVRAICVMKSFLIFRRWKSHSPLQIQLHLSHPITTSCSIPWCHLC

B.					
Gene	cMS	MW(w.t.)	MW(mut)	a.a.	Mutant neo C-termin us (−1 DNA base pair frameshift)
ASTE1	A11	77091	74956	32	GRRNRIPAVLRTEGEPLHTPSVGMRETTGLGC
PTHLH	A11	23942	22251	11	RKTTEEHIICN*
MARCKS	A11	31553	15650	11	RSAFPSRSLSS
ACVR2A	A8(2)	57847	49559	4	RGLF
C4orf6	T10	10498	9166	58	SCQLNLGRKEHAKIFTFFFQLDTMDGNPGELTLELQTLQIKQSQNALLPAGPLTQTPV
AIM2	A10	38953	40387	13	HREVKRTNSSQLV
TCF7L2	A9	65288	53863	22	SAFATYKVKAAASAHPLQMEAY*
EPHB2	A9	117488	120698	62	REWEKRKQILGGGGKYKEYFLKRILIRKAMTVLAGDKKGLGRFMRCVQSETKAVSLQLPLGR
TFE3	G8	61518	56928	43	DLPRMLPISSPLHRPQMPFWTCTFPATTWGTWETPSTWGWRTF
RGS12	C8	149587	145966	48	RSLPAGPSALPSPPSPSRRRAPPRSGRGSLRKWRPGASRRWRMSTWPS
TTK	A9	97069	100952	38	GEKNDLQLFVMSDTTYKIYWTVILLNPCGNLHLKTTSL
PA2G4	A8	43785	43230	15	RRPPRLQRMPPVGKH
AIM2	A6	38953	37115	6	MEKNYS
CREBBP	C5	265351	208941	8	LPSPNPHP
EP300	C5	264161	187834	8	TMTTKWRN

Mutant sequence commences from 1st difference compared to wild type protein after the mutated cMS. a.a.: amino acids in mutant sequence. MW: Predicted molecular weight of wild type (w.t.) and mutant (mut) protein in Daltons.

*alternative transcripts for these genes generate mutant transcripts with a different reading frame, encoding for different mutant C-termini.

### Detectable MSI-derived mutant proteins are expressed by NMD-resistant transcripts

**Figure 1:**
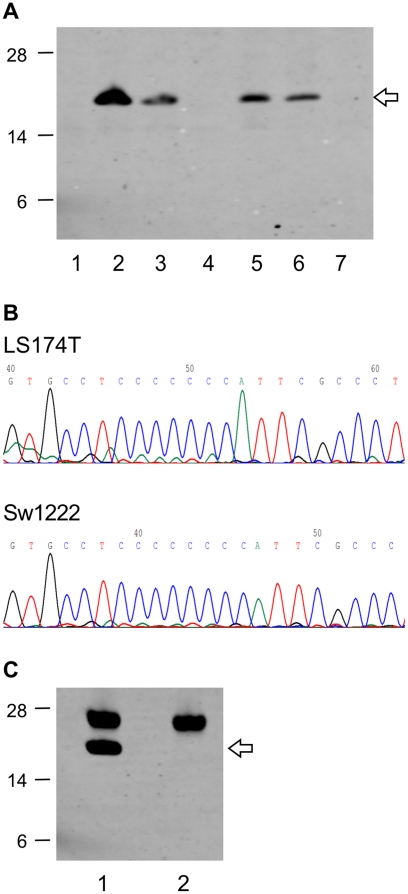
Expression of normal BAX protein, but not mutant BAX protein by MSI-High cell lines. (**A**) Normal BAX protein (21 kDa, arrow), but no mutant BAX protein (predicted 6.4 kDa) was detected in Western Blot analyses on whole cell lysates of colon cancer cell lines. Cell lines with BAX G8 cMS mutation status: 1. LoVo (−1, +1), 2. SW480 (wt), 3. HCA7 (−1, wt), 4. LS174T (−1), 5. HCT116 (−1, wt), 6. HeLa (wt), 7. LIM1215 (−1). (B) Homozygous −1 base mutation in G8 cMS in LS174T cell line (top) compared to non-mutated cell line, SW1222 (bottom) (reverse DNA sequencing). (C) Normal BAX protein (arrow), but no mutant BAX protein detected by immunoprecipitation: 1. HeLa (wt), 2. LS174T (−1). IgG light chain at 25 kDa.

To detect expressed mutant proteins, we purchased antibodies to the conserved N-terminal portion of selected proteins. We initially studied a selection of commonly mutated genes reported to be potential “target genes”[1]. Western blot experiments on whole cell lysates and immunoprecipitates of the genotyped cell lines identified protein bands corresponding to the expected molecular weight for normal BAX (Figure 1), MSH3 and CASP5 proteins (unpublished data) in cell lines with non-mutated alleles of these genes. However, no mutant protein bands were detected for any of these genes. In the case of BAX we generated polyclonal rabbit antibodies to a synthetic peptide corresponding to the mutated C-terminus. These antibodies showed specific binding to synthetic mutant peptide in Biacore, ELISA and Western blot assays, but did not detect a mutant BAX protein band in whole cell lysates or immunoprecipitates of BAX mutated cell lines (unpublished data). Mutations in BAX, MSH3 and CASP5 all encode for NMD-sensitive (NMD-S) mutant transcripts; mutant BAX and MSH3 are known to be susceptible to degradation[27]. NMD-sensitivity could account for our inability to detect any mutant proteins arising from these three genes, though abnormal protein folding with loss of the antibody epitopes might also be a factor.

**Figure 2:**
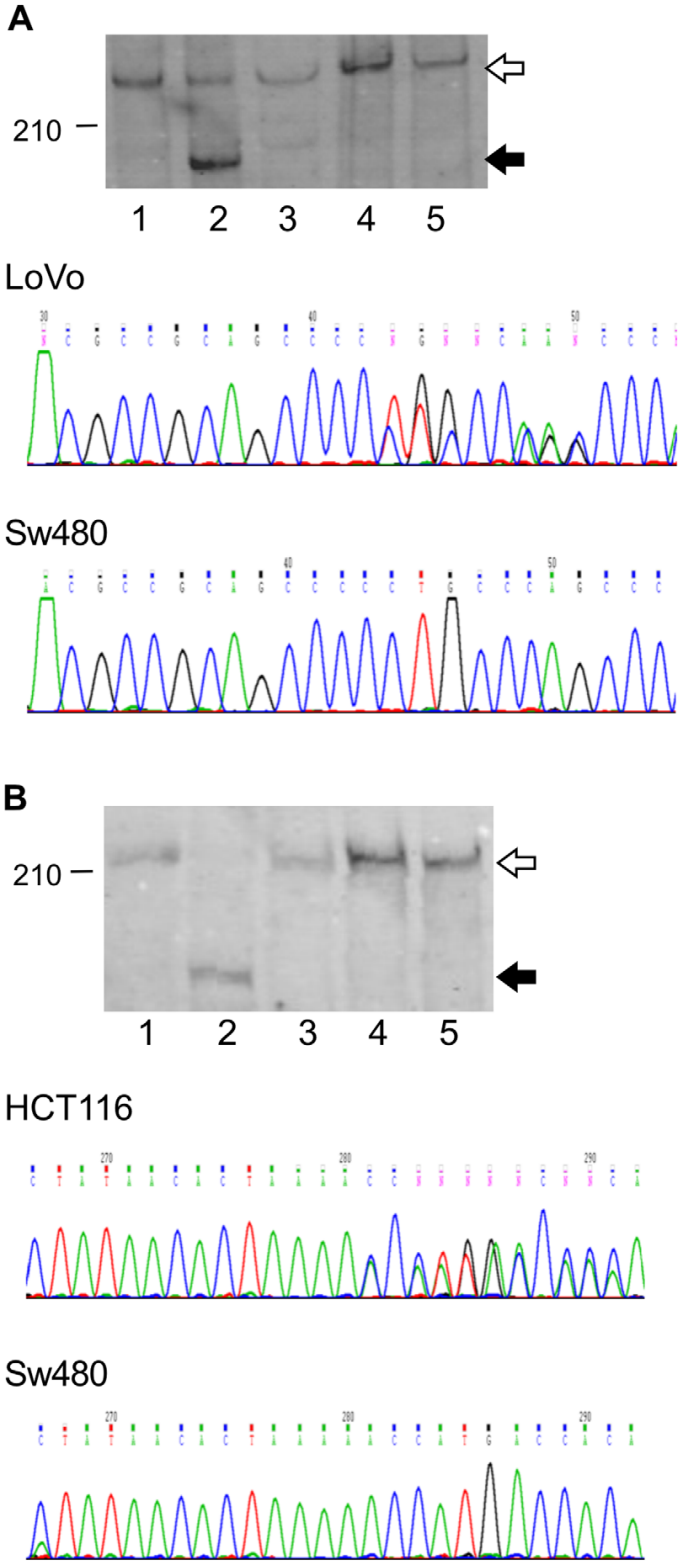
Expression of mutant CREBBP and EP300 proteins, arising from NMD-resistant mutant transcripts. Normal proteins (white arrow) and mutant proteins (black arrow) were detected in Western Blot analyses on whole cell lysates for (A) CREBBP and (B) EP300. (A) CREBBP C5 cMS: 1. SW480 (wt), 2. LoVo (−1, wt), 3. HCT116 (wt), 4. LIM1215 (wt), 5. LS174T (wt). MW (kDa): wt 265, mut 209. (B) EP300 A5 cMS: 1. LoVo (wt), 2. HCT116 (−1, wt), 3. HCA7 (wt), 4. LIM1215 (wt), 5. LS174T (wt). MW (kDa): wt 223, mut 187.

We therefore sought to detect mutant proteins arising from NMD-resistant (NMD-R) transcripts. Previously published Western blot data showed potential mutant protein bands of the CREBBP and EP300 genes, each arising from a heterozygous (−1, wild type) frameshift mutation of a 5 mer cMS repeats in the last exon, detected in the LoVo (CREBBP) and HCT116 (EP300) cell lines respectively[22]. We confirmed the presence of these mutations in LoVo and HCT116 cell lines by PCR and DNA sequencing. These cMS were not mutated in any of the other MSI-High or MSS cell lines. Bands corresponding to the expected molecular weights of the normal CREBBP and EP300 protein were consistently detected in Western blot analyses of non-mutated cell lines. We detected abnormal protein bands corresponding to the expected molecular weights of mutant CREBBP and mutant EP300 in the LoVo and HCT116 cell lines respectively in Western Blots on whole cell lysates (Figure 2) and on immunoprecipitates (Figure S1). Our blot matched the previously published data for EP300[22]. The prior CREBBP data matched a region of non-specific bands in our blot, but protein bands of appropriate molecular weight were also present in our blots. The CREBBP bands were also detected in experiments where one antibody was used for an immunoprecipitation and an antibody to a different CREBBP epitope was used for detection.

### Validation of the mutant proteins

**Figure 3:**
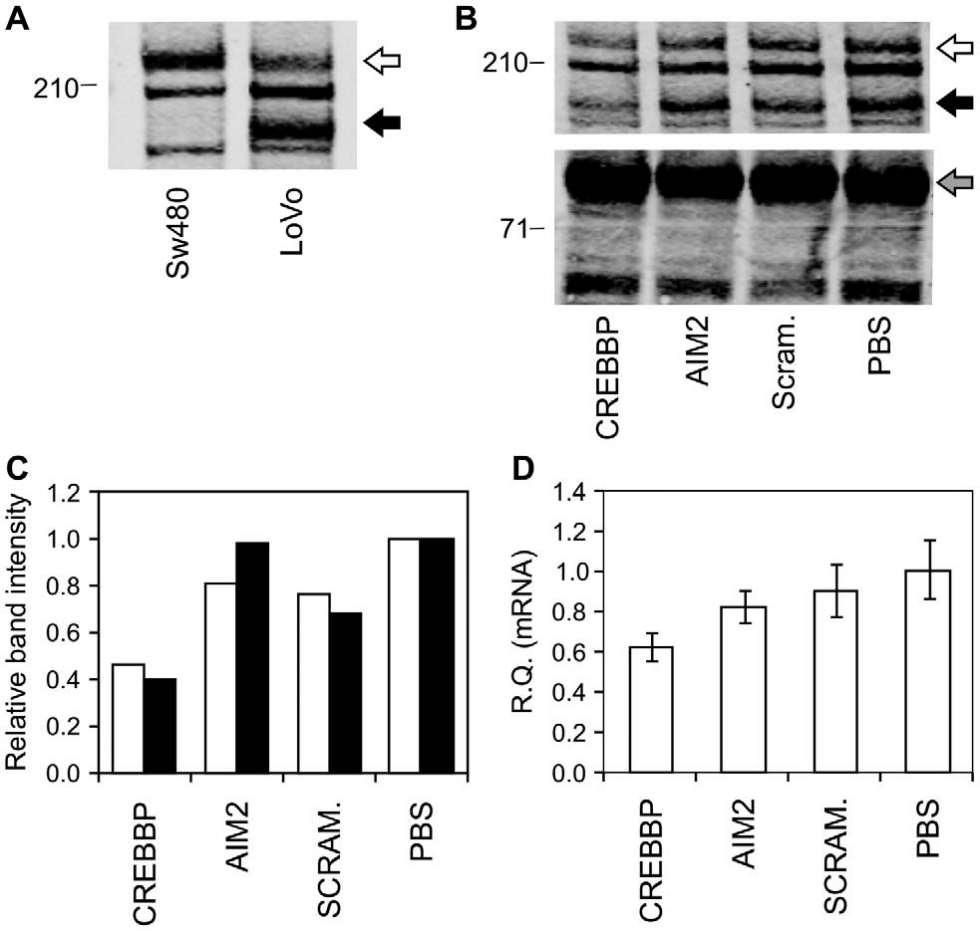
Validation of mutant CREBBP protein. Specificity of the mutant protein band was confirmed by gene-specific knockdown in transfectants of siRNA targeting CREBBP. (A) Detection of mutant protein band (black arrow) in whole cell lysates of LoVo cell line, but normal protein only (white arrow) in non-mutated SW480 cell line. (B) Western Blot of LoVo whole cell lysates from siRNA SMARTpool transfectants show decreased expression of normal (white arrow) and mutant (black arrow) CREBBP protein band in CREBBP-targeted lysates (lane 1). Beta-catenin loading control (grey arrow) 92 kDa. (C) Relative intensity of normal (blue) and mutant (red) CREBBP protein bands compared to untransfected cells (PBS). (D) Decreased CREBBP mRNA expression in CREBBP siRNA transfectants (left lane) relative to untransfected cells (right lane).

**Figure 4:**
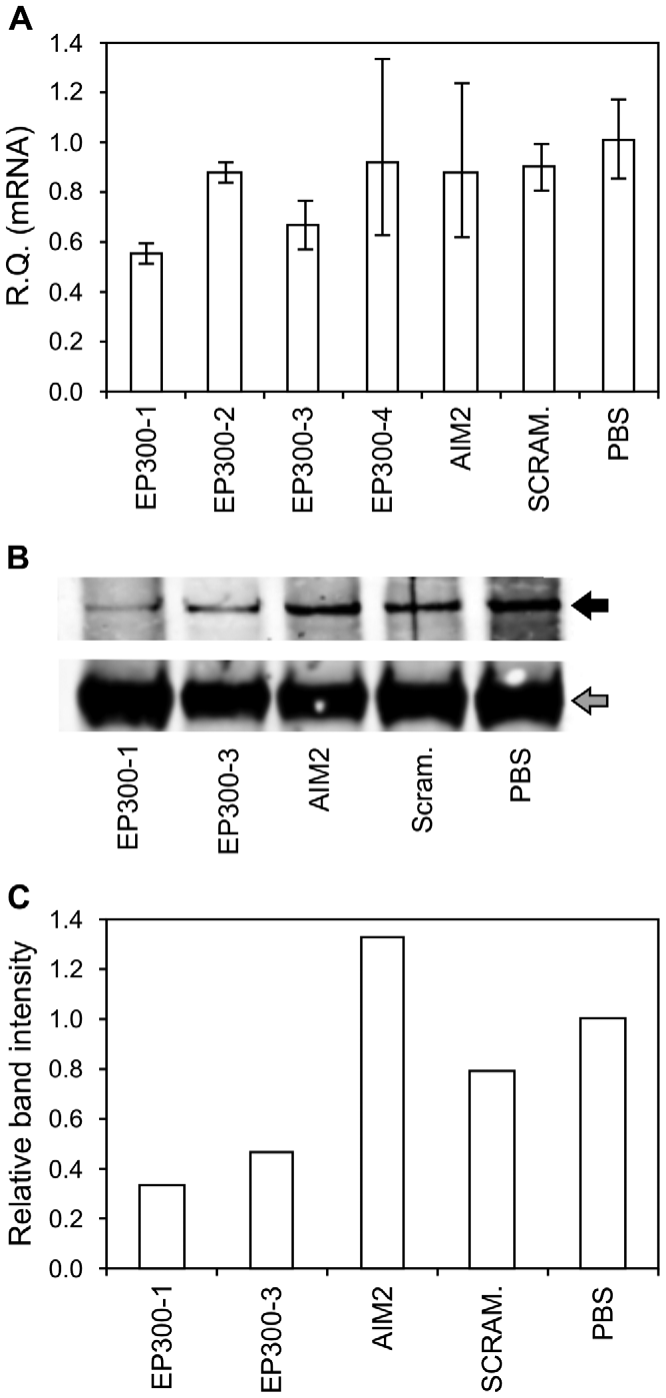
Validation of mutant EP300 protein. Specificity of the mutant protein band confirmed by gene-specific knockdown in transfectants of siRNA targeting EP300. (A) qRT-PCR data to assess siRNAs targeting EP300 (best knockdown using P300-1 and P300-3). (B–C) Decreased mutant EP300 protein detected in Western Blot analyses on immunoprecipitates of EP300 siRNA transfectants relative to untransfected controls (PBS).

To confirm the identity of the abnormal protein bands and prove that these represent genuine examples of the expected mutant proteins, we initially performed tandem mass spectrometry on immunoprecipitates of the cell lines. This technique confirmed the identity of the normal CREBBP and EP300 protein bands (Figure S1), thus validating the specificity of the antibodies used. The mutant proteins were not detected by this method, possibly due to co-immunoprecipitation of more abundant proteins of similar molecular weight such as MYH9 (unpublished data). The specificity of the mutant protein bands was instead confirmed by using small interfering RNA (siRNA) to perform gene specific knockdowns of the mutant CREBBP and EP300 proteins. Decreased expression of the mutant CREBBP protein relative to untransfected and non-targeted controls correlated with a significant decrease in targeted mRNA, confirmed by qRT-PCR (Figure 3). Knockdown of the mutant EP300 protein was similarly demonstrated in immunoprecipitates (Figure 4). These results confirm that the abnormal protein bands detected are mutated variants of the CREBBP and EP300 proteins. This finding indicates that certain MSI-derived mutant proteins are naturally expressed at detectable levels.

### Detection of additional potential mutant protein bands

**Figure 5:**
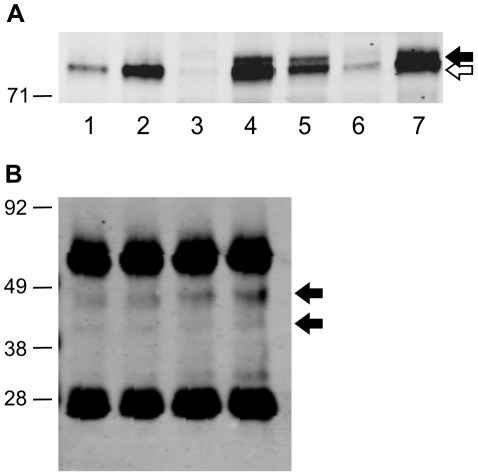
Potential mutant TTK and AIM2 proteins. (A) Western blot analyses on whole cell lysates identified a potential mutant TTK protein band (black arrow) in multiple mutated cell lines and normal TTK protein (white arrow) only in non-mutated lines: 1. LIM1863 (wt), 2. HCA7 (wt), 3. LIM1899 (−1, wt), 4. HCT116 (−1, wt), 5. LoVo (−1, wt), 6. LIM2537 (−1, wt), 7. LIM2551 (−1, wt). TTK MW (kDa): wt 97, mut 101. (B) Western blot analyses on immunoprecipitates of whole cell lysates identified an AIM2 protein band (black arrow) in all cell lines, including the homozygous mutant LoVo cell line: 1. SW480 (wt), 2. LoVo (−1), 3. HCT116 (−1, wt). 4, LS174T (wt). AIM2 MW (kDa): wt 39.0, mut 40.4.

In further Western Blot experiments (Figure 5), mutant TTK protein bands were detected in multiple mutated cell lines at slightly higher molecular weight (101 kDa) than non-mutated TTK protein (97 kDa), consistent with the predicted size (Table 3). Attempts at siRNA knockdown of mutant TTK were hindered by difficulty in resolving the mutant and normal protein bands. An AIM2 band was detected at about 46 kDa in Western blots on immunoprecipitates from whole cell lysates, consistent the size detected in previous studies using this antibody[28]. AIM2 protein was detected in multiple non-mutated cell lines and also in homozygous AIM2 mutant LoVo cells. The mutant and wildtype AIM2 proteins are of similar predicted molecular weights (39 kDa and 40 kDa respectively). Because NMD-resistant transcripts often have a cMS in the last exon near the C-terminus, mutant proteins will often be of similar size to normal proteins. This makes validation of these mutant protein bands by techniques used to confirm the CREBBP and EP300 bands more difficult.

Mutant protein bands were not detected for all NMD-resistant transcripts assessed. In the case of ACVR2A, protein bands were detected at the expected molecular weights for both normal and mutant protein, but in a non-specific manner that did not correlate with the mutation data. Multiple protein bands were detected in blots for TCF7L2, a gene with numerous alternatively spliced transcripts[29], but no mutant proteins were identified that correlated with the mutation data.

### Genome-wide search for NMD-resistant transcripts as a source of targetable proteins

Having established that NMD-resistant transcripts can give rise to expressed MSI-derived mutant proteins, we sought to identify additional cMS-containing genes likely to generate expressed mutant proteins in MSI-High colon cancers that could serve as therapeutic targets. Using a bioinformatic strategy, we performed a genome-wide search for cMS-containing genes based on criteria predicted to encode for NMD-irrelevant mutant transcripts[24], which are intrinsically NMD-resistant (Table S2). We identified 1511 non-redundant mononucleotide cMS repeats ≥7 mer in length (including hypothetical genes) predicted to generate NMD-resistant mutant transcripts. This compares with 17,654 mononucleotide cMS (≥6 mer) identified (irrespective of NMD status) in a previous study[17].

Genes were selected for cMS mutation analysis based upon selection criteria likely to be important to utility as a targetable protein. We limited the study to the 316 mononucleotide cMS repeats ≥8 mer in length, since there is a correlation between microsatellite length and likely mutation frequency. Significant mutation rates (>40%) have been reported for some 8 mer repeats[16]. Genes with short predicted neo-C-terminal protein sequences (<10 amino acids) were excluded, as these are unlikely to generate useful T cell epitopes for a range of common HLA types, as is required to overcome MHC restriction. Excluding hypothetical genes (prefix LOC or KIAA), 179 cMS-containing genes satisfied our selection criteria (Table S3). Evidence for protein expression in colon cancers of the shortlisted genes was also sought as an additional selection criterion to indicate likely expression of the mutant proteins. The Human Protein Atlas [30] includes data showing protein expression in colorectal cancers by immunohistochemistry. However, such data is unavailable for many genes, due to dependance of IHC upon the availability of suitable antibodies. Therefore, gene expression profiling data was also used to identify genes with the highest mRNA expression levels based upon data from two studies [31], [32] of MSI and MSS colon cancers (Figure S2).

Selected genes (Table 4) were screened for cMS mutations in a panel of 5 MSI and 1 MSS cell lines and a subsequent panel of 6 MSI cell lines was tested if the initial screen revealed any cMS mutations. Three genes (SFRS12IP1, MED8 and ASXL1) were mutated in 6/11 (55%) of MSI-High cell lines and two genes (FBXL3 and RGS12) were mutated in 5/11 (45%). Several genes with lesser mutation rates were also identified (Table S4).

**Table 4:** NMD-R C-terminal peptide sequences from genome-wide search selected for mutation analysis.

Gene	cMS	MW(w.t.)	MW(mut)	a.a.	Mutant neo-C-terminus (−1 base pair frameshift)
SFRS12IP1	A10	18176	18253	66	GKGLTHPVPLKRTLQNKRNKNIRRKKRKKKKRVNQKKGNITKRKKRREKRKSILLHLIVLNSPESN
MED8	A9	32817	38899	61	MPTCSSGEAWACSPFHPLRSQLGFYLSNSHKVPYPLPHLIHTRLSQEVALPGRTMWERVFP
ASXL1	G8	165441	77046	57	VARVEVAAGPPMREVAEAAAVVMVVRPVATLSPGEARAPLESVRQIYSEHNYCRLIL
FBXL3	T9	48705	36034	24	YMKKNLTPSFAMKYLPPICTLGDQ
RGS12	A9	149587	132211	31	NIRKLIWTKQRSFLSLFPKLRATEQMTNVGC
RTKN2	A9	69301	59002	66	LKRQMGSSLLVSMKNPYHLLGPHSLMVTIKWSSRKRYCILQVSHYMMKKGKRDKLPFLLLINFHSA
RTN3	A8	27567	35071	66	RQNKYMETRNATVTKTPFNSYNVVTCTMKENTQCQLEPAFQAFFLIWCFLPSFPFNPQYQAQKLMD
SAFB2	C8	107469	111382	48	RTPTSPAATKSHSLRVFGWADALLNLVARVPSNLGDLFKSKVNPATML
TMEM97	A10	20847	41744	187	NEGNNHWPRVEMPTGWLLVGYNTRNTAQNPRLQQHLKHWQQCTRARWCQEPCQTLTFFHFFFFFKTVSLCCP…
TMEM97 (cont)	-	-	-	-	…GWSKGQWHDLGSLQPPPPGLKPSSLASQVARTTGVYQHVWLICFVFFVCVETGFCHVAQVGLERLGSSD…
TMEM97 (cont)	-	-	-	-	……LPTSVSLSAGITDVNHWAQPKPSPSKGTGMNRPIGLRVGKGVWARL
ANKRD49	A9	27289	11639	31	WKKTQADCFFGLLKKIGLPQCGDSFLKRPLT
VRK2	A8	58138	43564	20	STVREALSPVQHGKCRKRRN
EAPP	A8	32761	20412	54	RRNNTRFQQMTNYCMILKKITEIRPGLMHREGVTMVWDHRDHVNNSLFQIVMLS
NTAN1	A8	34676	40304	62	MKMACGKRSLLQEVKKHELPKKAPSWPDRPLVGLARIQIWILHLLGLRPPSLLSVFQMTFIK
RAPH1	C8	135250	86746	18	HLLLSLHPSLPKLPQNPL
CCKBR	T8	48417	40685	34	CVGCQFIVPTRGAPLMARVHTEHSRVLLSPSFTC
P4HB	A8	57114	57013	117	TSLWSSMPHGVVTANSWLPFGINWERRTRTMRTSSSPRWTRLPTRWRPSKCTASPHSSSFLPVPTGRSLITTGNA…
P4HB (cont)	-	-	-	-	…RWMVLRNSWRAVARMGQGMMTISRTWKKQRSQTWRKTMIRKL
RAB40C	C8	31303	36007	57	RTARGVTARSPSGDGRGRLCRCQEGSSWTLLAGRQASAAYVETVHTAASEAPGFPHT
ST8SIA6	A8	44834	34098	52	PYFWRTLQPMEMHFFFCQHFPSGPTRVPLSKYTTRSKSLKQDKRFYFSIPST
CIR	A9	52311	38786	50	YKGRKERKTSVQGITTVILKRRTSLRRESFMKNFLAVTITGKKPRKSPGS
KCNC1	A8	57940	54089	19	RSIFRGHRSWDLPIIVNLS
SP100	A8	100413	90992	39	RSSRIPMHPRGLLWPFSCSALSIAQKSKENILACPLMML
C15orf48	A9	9617	7122	16	IQNLGKLWTLLYLKSL
DYRK3	A8	65711	37630	34	ISFRVLASSWYASLPSPSCNLWMPSTKIRLFTAI
ERCC5	A9	133103	110374	64	NYGHCNSPLAFLTQLLPRPTSNPWWMTRRDPFCGGNLISTKLENFVSGISAGTERRQMNLCFLY
GPATCH4	A8	42053	31346	72	RKRKGGRKRRKKLQHLKGMMQMRSTQNMLSRTSEKARRRKGDIKKERSQMKERVQLKGMRRRTLQEQVGLGN
HOXB7	G8	24014	17050	98	AWRARARPASTRPAMGSSRVPSTCTARPLSRTSPGCVPATPPRRRAPRSRGTRTWRPRVTSGSTPGCEAQELTA…
HOXB7 (cont)	-	-	-	-	…NEAARPTPATRPWSWRKNFTTIAT
INTS8	A8	113084	117050	43	SFSKQWQNFTFKQLNFFNFYFLNNGLKINSIKRLSLYNTYVHN
RARRES3	A8	18178	23706	55	RQPEAATKSCVRSSCGGPSGDEPPPCLQQPDPRALSQAFSRSFPLFPSLAGKSMI
SF3B2	A8	100223	104095	58	NGKLSPRTAVGAARNIRSSSFRSPHTSPFFGPTSGCLGFTQEPPLPQFPRTCHFMFLF
TCERG1	A8	123896	114436	40	SLRKILDVLSSPPVTGKNKENLKNISETNISQPKLTSGRF
WHSC1	C8	152252	154227	48	QSQGSRRGRGGGGGAGGESQRANSARRPLGRIQGRCRAAGPACGRGRA
ZNF133	A8	73315	20485	120	NVHRQPVQIQSQSSTSILSALRVSPVRNSPCSMCCVIIPPGSSHACVQKVTSSLGIRAQGTRRSSNKPLRGDPGV…
ZNF133 (cont)	-	-	-	-	…IKQKVLREKVPCLCLEEPRKGLWERSPGHPRGSQSALGTASEGWS

### *In silico* epitope analysis for NMD-resistant transcript derived mutant proteins

To be useful as therapeutic vaccine/immunotherapy targets, the mutant proteins identified need to generate CTL epitopes for common HLA types. *In silico* analysis using the publicly available software such as SYFPEITHI has been previously identified new CTL epitopes that were subsequently validated in functional assays[26]. To identify which MSI-derived mutant proteins are likely to be immunogenic, we assessed mutant proteins arising from NMD-resistant transcripts for potential HLA-A*0201 epitopes. HLA-A*0201 is the most common HLA type, present in about 50% of the population[33]. SYFPEITHI scores are based upon binding motifs common to known naturally occurring epitopes. According to the SYFPEITHI scoring guidelines, a naturally expressed epitope should be amongst the top 2% of all peptide scores assessed in 80% of cases[26]. We assessed mutant C-termini arising from common NMD-R mutations (Table S5) and the top 2% of HLA-A*0201 SYFPEITHI binding scores generated from the mutant proteins were determined (Table 5). The top-ranking peptides had SYFPEITHI binding scores ranging from 23–31. These scores are comparable to the binding scores of 135 known HLA-A*0201 CTL cancer epitopes[26], including the 4 published MSI-derived epitopes, which have a median score of 24 and a mean of 23.4 (Table S6).

**Table 5:** HLA-A*0201 SYFPEITHI [26] scores of (A) top ranking NMD-R derived epitopes compared with (B) published MSI-derived epitopes.

A				
GENE	HLA-A*0201 epitopes	SCORE	Reported (%)	This study (%)
RAPH1	S L H P S L P K L	31	No data	18
RAPH1	L L L S L H P S L	28	No data	18
RAPH1	H L L L S L H P S L	25	No data	18
RAPH1	S L P K L P Q N P L	23	No data	18
NTAN1	G L R P P S L L S V	30	No data	18
NTAN1	L L Q E V K K H E L	27	No data	18
NTAN1	G L A R I Q I W I L	27	No data	18
NTAN1	H L L G L R P P S L	27	No data	18
NTAN1	K M A C G K R S L	25	No data	18
NTAN1	L L G L R P P S L	25	No data	18
NTAN1	Q I W I L H L L G L	25	No data	18
NTAN1	R I Q I W I L H L	23	No data	18
NTAN1	R I Q I W I L H L L	23	No data	18
TMEM97	S L S A G I T D V	28	11	36
TMEM97	S L Q P P P P G L	27	11	36
TMEM97	W L I C F V F F V	27	11	36
TMEM97	S L A S Q V A R T	24	11	36
ASXL1	V M V V R P V A T L	27	No data	55
ASXL1	M V V R P V A T L	23	No data	55
EPHB2	I L I R K A M T V	27	41	36
EPHB2	V L A G D K K G L	25	41	36
EPHB2	I L I R K A M T V L	24	41	36
TTK	L L N P C G N L H L	26	27	70
TTK	I L L N P C G N L	25	27	70
TTK	N L H L K T T S L	25	27	70
TTK	K I Y W T V I L L	24	27	70
TTK	V M S D T T Y K I	23	27	70
SFRS12IP1	S I L L H L I V L	26	No data	55
RGS12 (A9)	S L F P K L R A T	25	No data	46
RGS12 (C8)	S L P A G P S A L	25	28	18
RTKN2	H L L G P H S L M V	25	No data	36
RTKN2	L L G P H S L M V	25	No data	36
EAPP	M I L K K I T E I	25	No data	18
EAPP	C M I L K K I T E I	24	No data	18
EAPP	L M H R E G V T M V	24	No data	18

B				
GENE	HLA-A*0201 epitopes	SCORE	Reference	
FTO (U79620)	TLSPGWSAV	25	[14]	
OGT	SLYKFSPFPL	23	[13]	
CASP5	FLIIWQNTM	20	[12]	
TGFBR2	RLSSCVPVA	19	[10], [11]	

Based upon available reported cMS mutation rates for EPHB2, TTK and RGS12 (C8), a vaccine including these three mutant proteins will include at least one probable HLA-A*0201 epitope for 69% of patients (1−(1−41%)x(1−27%)x(1−28%)). Further HLA-A*0201 epitopes were identified for several genes (TMEM97, ASXL1, SFRS12IP1, RGS12 (A9), RTKN2) with common mutations in this study, indicating that also targeting these genes could broaden HLA-A*0201 coverage. Some rare mutations (e.g. NTAN1, RAPH1) also produce immunogenic epitopes that would not provide broad population coverage, but may also be worth targeting in selected patients.

## Discussion

Microsatellite instability gives rise to predictable frameshift mutations involving the cMS of many genes. Mutant proteins arising from these mutations are a logical source of tumour-specific antigens that could account for both the presence of increased TILs and the better prognosis associated with an MSI-High phenotype. This concept is supported by a recent finding of a correlation between immune infiltrate density and the number of cMS frameshift mutations detected in a set of MSI-High colon cancers[34]. We have validated at least two examples (CREBBP and EP300) of naturally expressed mutant proteins in MSI-High colon cancer cell lines, both arising from NMD-resistant transcripts. These findings confirm that NMD-resistant transcripts can generate naturally expressed MSI-derived mutant proteins[34], [35]. The cMS repeats in CREBBP and EP300 are short and unlikely to be mutated in a high proportion of MSI-High tumours. However, it is likely that some common NMD-resistant mutations such as TTK will also generate targetable mutant proteins in MSI-High cancers. Our genome-wide strategy to identify immunogenic NMD-resistant transcripts has identified five novel cMS mutations (SFRS12IP1, MED8, ASXL1, FBXL3, RGS12) in at least 45% of eleven MSI-High colon cancer cell lines tested. Mutation analysis in a larger series of primary MSI-High colon cancers will enable the mutation frequencies of these potential therapeutic targets to be more accurately determined.

*In vitro* studies have identified CTL responses to synthetic mutant peptides arising from common cMS mutations, using peripheral blood mononuclear cells from patients with MSI colon cancers[10], [11], [12], [13], [14], [36] and intriguingly, also in healthy individuals with HNPCC[15]. These studies have shown that tumour-specific mutant proteins can stimulate *in vitro* T cell immunity and support the concept of a vaccination strategy. The published HLA-A*0201 CTL epitopes for TGFBR2[10], [11], CASP5[12], OGT[13] and U79260(FTO)[14] all arise from NMD-sensitive mutant transcripts. NMD-inhibition experiments have shown that mutant TGFBR2 is one of the most upregulated transcripts, with 10-fold increased mRNA expression in one study[27], [37]. The T cell responses conflict with the expected impact of NMD upon mutant protein expression. A transfection study showed that mutant TGFBR2 protein is detectable in transfectants of mutant cDNA (NMD-resistant), but not mutant gDNA (NMD-sensitive)[35]. We did not detect mutant proteins from NMD-sensitive transcripts (BAX, CASP5 and MSH3). However, NMD is a translation-dependant process[24] and low-level mutant protein expression from the initial round of translation may be sufficient to stimulate immune responses. CTL can show exquisite sensitivity to a specific peptide/HLA ligand, with as few as 3 target peptide/MHC complexes being sufficient for CTL-specific cell lysis *in vitro* [38]. Humoral responses to mutant TGFBR2 protein have been identified by ELISA, but only in a minority (10%) of MSI-High colon cancer patients[39].

A benefit of NMD-resistant transcripts as a source of targetable mutant proteins is that stably expressed tumour antigens can be cross-presented to the immune system by dendritic cells to stimulate CTL and T helper cell responses[40]. Cross-presentation is likely to result in superior anti-tumour immunity and NMD-resistant transcripts as a source of targetable mutant proteins. A fusion-gene assay reported a significant correlation between the expression of transfected MSI-derived mutant proteins and cross-presentation of co-expressed ovalbumin antigen [18]. For genes with negligible detectable protein, some moderate to strong CTL responses were detected, but without cross-presentation[18]. A limitation of the transfection assay is that mutant cDNA constructs were used and therefore the experiments did not evaluate the impact of NMD upon mutant protein expression. Our approach overcomes this through direct detection of mutant protein expression. A modified transfection assay requiring natural splicing of the C-terminus could also identify biologically relevant vaccination targets.

NMD-inhibition experiments have identified some “NMD-escape” transcripts which partially resist degradation due to NMD[27],. MBD4 is predicted generate an NMD-sensitive transcript, but a faint abnormal protein band has been reported in Western blot analyses of MBD4 mutated cell lines. This may represent mutant MBD4 protein expression due to NMD-escape[23]. NMD-escape transcripts may be a useful source of protein targets, but these are unable be predicted and further work is needed to determine which NMD-escape transcripts produce targetable mutant proteins. Strategies that interfere with NMD without inhibiting protein translation should cause a generalised upregulation in NMD-sensitive mutant transcripts, unmasking a range of tumour antigens. This approach could complement a vaccine targeting MSI-High tumours by broadening the range of available immune targets beyond NMD-resistant transcripts. NMD-inhibition by siRNA targeting of NMD regulatory genes SMG1 or UPF2 has recently been shown capable of inducing protective *in vivo* immune responses to tumour cells transfected with an NMD-sensitive target antigen[41].

Our confirmation of mutant protein expression by MSI-High tumours may have important clinical applications. A vaccine targeting commonly expressed mutant proteins has been proposed as a promising strategy for the treatment of MSI-High tumours [15], [18], [34], [42]. With sufficient targets, vaccination may be feasible provided that the mutant proteins are expressed, antigenic epitopes are generated and antigen presentation is functional. Further work is needed to determine which mutant protein immune responses can confer a survival benefit to patients with MSI-High tumours. Mutant proteins secreted into the serum could serve as useful biomarkers for the early detection of MSI-High tumours, particularly in the HNPCC affected population. Such biomarkers would be a useful adjunct to colonoscopic screening, since MSI-High colorectal cancers have a propensity for interval adenomas and tumours developing in the period between screening colonoscopies[43], [44]. The common, predictable and tumour-specific nature of MSI-derived mutant proteins makes them ideal candidates as therapeutic targets or diagnostic markers.

## Materials and Methods

### Cell lines and culture

LIM colorectal cancer cell lines were established from tumour biopsies at the Ludwig Institute for Cancer Research (LICR) Melbourne Branch as described previously: LIM1215[45], LIM1899[46], LIM1863[47], LIM2463[48] and (LIM2099, LIM2405, LIM2408, LIM2537, LIM2550 and LIM2551) [49]. The LIM cell lines were established from both sporadic and familial colorectal cancers[49] and can be obtained from CellBank Australia (www.cellbankaustralia.com) or from the LICR Melbourne Branch on completion of a standard academic materials transfer agreement. SW1222 colorectal cancer cells were provided by the Ludwig Institute for Cancer Research, New York Branch, (New York, NY) and HCA7 colorectal cancer cells were obtained from the European Collection of Cell Cultures (Porton Down, UK). HCT116, LS174T, SW480, LoVo and HT29 colorectal cancer cell lines and HeLa cervical cancer cells were obtained from the American Type Culture Collection (Rockville, MD). Cells were cultured at 37°C and 5% CO_2_ in RPMI 1640 media (Invitrogen) supplemented with 10% foetal calf serum (FCS), 8 U/ml insulin, 0.5 mg/ml hydrocortisone, 1×10^−2^ mg/ml thioglycerol, 20 µg/mL streptomycin sulfate and 12 µg/mL benzylpenicilin or in DMEM media (Invitrogen) supplemented with 10% FCS, 20 µg/mL streptomycin sulfate and 12 µg/mL benzylpenicilin.

### Assessment for MSI by immunohistochemistry (IHC)

Cell blocks of each cell line were prepared from 5×10^7^ cells, rinsed and resuspended in 5 mL PBS, centrifuged into a pellet, fixed in 10% neutral buffered formalin and embedded in paraffin. Sections 3 µm thick were prepared using Superfrost Plus Adhesion slides (Thermo Scientific). IHC for DNA mismatch repair proteins was performed on the sections using Vision Biosystems Bond-Max automated IHC stainer, with on-board antigen retrieval using Bond Epitope Retrieval Solution 2 and polymer-based peroxidase staining using Bond Define Detection kit DS9713 and the following primary antibodies: MLH1 (Novocastra: NCL-L-MLH1), MSH2 (Oncogene: NA27), MSH6 (BD Transduction Laboratories: 970220-150) and PMS2 (BD Pharmingen: 556414). An anatomical pathologist (DSW) interpreted the IHC stained sections. Cell lines with nuclear staining for DNA MMR proteins were interpreted to have a normal expression pattern (likely MSS) and cell lines lacking nuclear expression of one or more DNA MMR proteins were interpreted as having MSI.

### Mutation analysis of cMS

Cell lines were tested for frameshift mutations in a selection of cMS reported to be mutated in MSI-High colon cancers by DNA sequencing (TGFBR2, BAX, CASP5, TCF7L2, TAF1B, MSH3, IGF2R, CREBBP, EP300, MARCKS, AIM2, ACVR2) or by fragment analysis (ASTE1, PTHLH, C4orf6, EPHB2, TFE3, TTK, PA2G4, KCNC1, SP100, FBXL3, MED8, RGS12, CIR). Genomic DNA was extracted from the cell lines with Qiagen DNeasy Blood & Tissue Mini Kit (Qiagen) using standard protocols. PCR primers (Figure S7) were designed to flank the cMS of interest, yielding amplimers 100–800 base pairs in length (for DNA sequencing) or 90–200 base pairs in length with FAM tags located at the 5′ end of the 5′ oligonucleotides (for fragment analysis). PCR reactions were performed in 25 µL total volume, with 100 ng gDNA, 2.5 µL 10× reaction buffer, 1 mM MgCl_2_, 100 µM dNTPs, 200 µM of each primer and 0.5 U Taq DNA polymerase, using the following standard conditions: initial denaturation at 94°C for 3 min, 35 cycles (denaturation at 94°C for 30 sec, annealing at 58°C for 30 sec and primer extension at 72°C for 1 min). A final extension step was performed at 72°C for 10 min. PCR fragments were resolved by agarose gel electrophoresis with ethidium bromide and visualised with ultraviolet light. Gel extractions were performed to purify PCR fragments (Qiagen) prior to DNA sequencing, using 5 ng DNA per 100 base pairs of template and 3.2 pmol of forward or reverse primer in 10 µl ddH2O (Wellcome Trust Sequencing Centre, Monash Medical Centre, Victoria, Australia). DNA sequencing data was analysed using Chromas Version 2.33 (Technelysium) and assessed for cMS frameshift mutations. DNA sequencing data from MSS cell lines were used as controls to indicate the degree of PCR slippage artefact affected the normal cMS sequences. For fragment analysis, PCR products were diluted based upon assessment of the intensity of the bands detected in the DNA agarose gel, loaded into 96 well plates with 2 µL sample, 9.75 µL formamide and 0.25 µL LIZ size standard per well for analysis. Samples were denatured at 95°C for 3 minutes, incubated on ice for 1 minute and loaded into a sandwich for analysis. Data was analysed using Gene Mapper 4.0 software (Applied Biosystems). Samples from MSS cell lines were used as controls to indicate the normal fragment size and pattern of non-mutated cMS.

### Prediction of mutant proteins arising from frameshift mutations

Genetic sequences were accessed from Ensembl[50] (latest release 58 – May 2010) and transcripts were assessed for the presence of the described cMS. Normal protein sequences and mutant protein sequences expected to arise from a frameshift mutation of −1 DNA base pair were determined for each cMS-containing transcript using ExPasy Translate Tool and the predicted molecular weight of the normal and mutant proteins were calculated with the ExPasy Compute pI/Mw Tool (Swiss Institute of Bioinformatics)[51].

### Western blotting

Whole cell lysates were prepared by incubation (15 min) of 10^6^ cells per 50 µL ice-cold RIPA lysis buffer (20 mM Tris-HCl (pH 7.2), 150 mM NaCl, 1 mM EDTA, 10% glycerol, 1% Triton X-100, 0.5% deoxycholate, 0.1% SDS, 1 mM DTT, 1× complete protease cocktail (Roche)). Cell debris was pelleted by ultra-centrifugation (10^5^ rpm, 30 min, 4°C). Immunoprecipitates were prepared by incubation for 2 hours of the lysates with Protein A-sepharose beads pre-coupled to primary antibodies (Table 6A). Whole cell lysates or immunoprecipitates were resuspended in sample buffer (2% SDS, 20% glycerol, 0.1 M TRIS pH 6.8, 50 mM TCEP bond breaker (Pierce), 0.004% bromophenol blue), boiled (5 min) and size-fractionated by SDS-PAGE using the Mini Protean system (BioRad, USA) and a 3–8% Tris-Acetate gel (NuPAGE Tris-acetate running buffer, Invitrogen, USA) for larger proteins (>150 kDa) or 4–12% Bis-Tris gel (NuPAGE MES running buffer, Invitrogen, USA) for smaller proteins. Gels were transferred (100 V, 4°C, 3 hrs) to a PVDF membrane (Millipore) in transfer buffer (25 mM TRIS, 200 mM glycine, 10% methanol (v∶v)), blocked overnight (5% skim milk powder, 0.02% azide), incubated with the primary antibody (1 hr), washed in PBS with 0.1% Tween-20 (PBST), incubated with the appropriate secondary antibody (Table 6B) for 1 hour and washed again. Membranes were imaged using the Odyssey Infrared Imaging System (LI-COR Biosciences).

**Table 6:** Primary antibodies (A) and secondary antibodies (B) used in western blot +/− immunoprecipitation experiments.

A. Primary antibodies.					
Antigen	Product (clone)	Species	Epitope	Dilution	Manufacturer
AIM2	(3B10)[28]	Mouse mAb	a.a. 1–186	1/10	Peter MacCallum Cancer Centre*
BAX	B8554 (2D2)	Mouse mAb	a.a. 3–16	1/2000	Sigma-Aldrich
BAX	ab7977	Mouse pAb	a.a. 11–30	1/100	Abcam
CASP5	ab40887 (EP876Y)	Rabbit	N-terminus	1/4000	Abcam
CREBBP	sc-689	Rabbit	N-terminus	1/200	Santa Cruz
CREBBP	MAB1133	Mouse	Unknown	1/200	Chemicon
CTNNB	610153	Mouse	a.a. 571–781	1/5000	BD Transduction Laboratories
EP300	sc-584	Rabbit	N-terminus	1/200	Santa Cruz
EPHB2	sc-28980 (H-80)	Rabbit pAb	a.a. 255–334	1/100	Santa Cruz
MSH3	611390 (50)	Mouse	a.a. 136–349	1/200	BD Transduction Laboratories
TTK	N1 (sc-56968)	Mouse mAb	a.a. 3–856	1/50	Santa Cruz

B. Secondary antibodies.					
Antigen	Antibody name	Species	Dilution	Manufacturer	
Mouse Ig	Anti mouse Ig odyssey 680 labelled	Donkey	1/10000	Li-Cor	
Rabbit Ig	Anti rabbit Ig odyssey 680 labelled	Goat	1/5000	Li-Cor	

*3B10 Hybridoma supernatant kindly donated by Dr Ricky Johnstone, Peter MacCallum Cancer Institute.

### RNA interference (RNAi) assays

Transient transfectants of cell lines were prepared using individual or SMARTpool preparations of small interfering RNA (siRNA) (Thermo Fisher Scientific), targeting the genes of interest. Transfectants of non-targeting (scrambled) siRNA and no siRNA (PBS only) were used as negative controls. Lipofectamine (7.5 µl) incubated with 250 µl OPTI-MEM media (Invitrogen) per transfection for 7 min was combined with siRNA (50 pmol in 250 µl OPTI-MEM per transfection) for 20 min at RT and added to cells pre-plated in 2 mL antibiotic-free OPTI-MEM media (6 well plates, LoVo 15×10∧5/well, HCT116 10×10∧5/well). After incubation overnight the siRNA-containing media was removed and replenished. At 24 hours post-transfection cell samples were split, one-third into fresh 6 well plates with 2 mL media/well for Western blot analysis and the remainder into 10 cm dishes and resuspended in 10 mL media/dish for qRT-PCR analysis. At 72 hours cells were harvested, counted using Countess Automated cell counter (Invitrogen) and lysed. Samples for Western blot analyses were stored at −80°C until ready for analysis. Specificity of candidate normal and mutant protein bands in western blots was confirmed by detection of decreased protein band intensity in lysates of cells transfected with siRNA targeting the gene of interest relative to negative controls. Blots were probed for a non-targeted gene (β-catenin) as a loading control to enable the relative “knock-down” to be calculated.

Extracts of total RNA for qRT-PCR were prepared from transfectants using RNAspin Mini RNA isolation kit (GE Healthcare) using standard protocols at room temperature. RNA concentration and quality (A260/280 ratio) was assessed with a NanoDrop ND-100 Spectrophotometer (Thermo Scientific). RNA samples were stored at −80°C until ready for analysis. Samples were digested using RNase free DNase and converted to cDNA with an RT kit (Applied Biosystems). Residual RNA was digested with RNAase. Serially diluted cDNA samples were analysed in duplicate by qRT-PCR with an ABI 7300 Real Time PCR instrument (Applied Biosystems), using Taqman (ABI) Master Mix, primers/probes for the gene of interest and for a control gene (GAPDH). No primer/probe, no RT enzyme and no cDNA negative controls were included and a standard curve was generated from the data. Fold-change in transfectant mRNA expression levels relative to untransfected cells was evaluated using the comparative C_T_ (ΔΔC_T_) method, expressed as a range incorporating the standard deviation of ΔΔC_T_. Gene-specific knockdown in siRNA transfectants was confirmed by detecting a significant decrease in mRNA levels of transfectants relative to untransfected cells.

### Nano-Liquid Chromatography-Mass Spectrometric Analysis

Immunoprecipitates of cell lysates (5×10^8^) were produced lysed and size fractionated by SDS-PAGE as described above. Gels for mass spectrometry analyses were stained (1 hr) with Coomassie R-250 (Pierce) and de-stained (>1 hr) in distilled water. Gel sections (1.5 mm) were excised with a scalpel, extensively washed in deionized water and digested with trypsin (0.25 µg) using a robotic workstation (MassPrep, Micromass). Tryptic digests were concentrated to ∼10 µL by centrifugal lyophilization (Savant) in preparation for Electrospray-Ion Trap (ESI-IT) tandem mass spectrometry (MS/MS) (LCQ-Deca, Finnigan). Digests (∼10 µL in 1% (v/v) formic acid) were transferred into 100 µL glass autosampler vials for injection and fractionation by nano-reversed-phase-HPLC (Agilent Model 1100 capillary HPLC) using a 150×0.15 mm I.D. RP-capillary column (Reprasil-Pur, C18-AQ, 3 µm, Dr Maisch, GmbH), linear 60 min gradient from 0%–100% Solvent B, flow rate of 0.8 µL/min at 45°C. Solvent A was 0.1% (v/v) aqueous formic acid and Solvent B was 0.1% aqueous formic acid/60% (v/v) acetonitrile. The capillary HPLC was coupled on-line to an ESI-IT mass spectrometer for automated MS/MS analysis of individually isolated peptide ions. Raw MS/MS spectra were extracted using Extract-MSN in Bioworks 3.1 (Finnigan, San Jose, U.S.A.). Parameters used to create the peak lists were: minimum mass 400; maximum mass 5000; grouping tolerance 1.5; intermediate scans 1; minimum group count 1; automated calculation of charge state; 30 peaks minimum per spectrum; peptide charge states 1+, 2+ or higher; ±2 Da peptide mass tolerance; ±0.5 Da MS/MS fragment mass tolerance. Parent ion masses were determined based upon the isotope cluster spacing in the zoom scan spectrum and individual spectra files (.dta file extension) were generated. Acquired MS/MS spectra were searched against the non-redundant protein subset database (Ludwig NR_subset version_Q407) using the Mascot™ search algorithm (v2.1, Matrix Science, U.K.). Searches were conducted with carboxymethylation of cysteine as a fixed modification (+58 Da), variable oxidation of methionine (±16 Da) and an allowance for up to 3 missed tryptic cleavages. Results containing possible peptide identifications by the Mascot algorithm were validated manually.

### Bioinformatic search for NMD-resistant mutant transcripts

Coding microsatellite (cMS) containing sequences likely to be NMD-resistant upon mutation were identified using a computer program written in the Python language (http://www.python.org) using the BioPython library of Python functions (http://biopython.org). Matched human genomic DNA, mRNA and coding DNA sequence (CDS) gene information was obtained from the RefSeq database (NCBI build 37.1; ftp://ftp.ncbi.nih.gov/genomes/H_sapiens/). The Python program processes each CDS entry, determines the coding DNA sequence and translates this sequence into a predicted amino acid sequence. Potentially erroneous CDS entries are discarded if they generate sequences lacking an initial methionine residue or a terminal stop codon. CDS entries generating sequences with a premature stop codon are also discarded. Retained CDS are checked for the presence of mononucleotide cMSs at least six bases in length (e.g. AAAAAA). One or two bases are deleted from the mononucleotide cMS and translated to generate amino acid sequences for the mutant proteins. If a premature stop codon in the frameshift mutated sequence is >55 bases upstream of the last exon-exon junction, this cMS is excluded from further consideration, as the mutant mRNA is likely to be NMD-sensitive. If a premature stop codon is in the last exon or within 55 bases of the last exon-exon junction, the cMS is retained, since the mutant mRNA is likely to be NMD-resistant. If no premature stop codon is generated, then frameshifted DNA sequence of the non-coding 3′ end of the corresponding mRNA entry is assessed for the presence of a stop codon. DNA sequences lacking a stop codon are discarded, since the entire sequence is unable to be characterized. Predicted mutant DNA and amino acid sequences are characterized by length and site of the cMS repeat, type of deletion (one or two repeat units) and the length, molecular weights and sequences of the normal and corresponding mutant proteins. The presence of any additional cMS in the genetic sequences is also identified to detect any lengthy 5′ cMS that may be susceptible to mutation and prevent some NMD-resistant transcripts from being generated. The program also assesses genes for dinucleotide cMS repeats using a similar algorithm.

### Identification of new target genes

Genes from the genome-wide search were prioritized for study based upon multiple criteria including susceptibility to frequent frameshift mutation, likely antigenicity and likely protein expression in colon cancer. A minimum mononucleotide cMS length of eight nucleotides was chosen, since mutation rates in our desired range (>30%–50%) have been reported for cMS of this length in some genes, but not for shorter cMS[16]. Genes with <10 amino acids of novel protein sequence were excluded, since these sequences are unlikely to generate a range of useful CTL epitopes. Immunohistochemical data regarding protein expression in colon cancer is available for some genes in the Human Protein Atlas[30]. In the absence of such data for many genes, gene expression microarray data were also used to assess which genes are highly expressed in MSI colorectal adenocarcinomas. Using two gene expression data sets that compared MSI with MSS colon cancers using the same Affymatrix chip (PMID: 17047040, GEO entry GSE4554[31], PMID: 19088021, GEO entry GSE13294[32]), we ranked shortlisted genes (NMD-resistant transcripts, ≥8 mer cMS, ≥10 amino acids of novel mutant C-terminus) by median gene expression values. Genes with either moderate to strong protein expression in colon cancer in the Human Protein Atlas resource or with median values in the top 50 scores by either gene expression study were considered for cMS mutation analysis. Gene expression data does not necessarily indicate relative expression between different proteins, since factors such as hybridization efficiency influence the microarray expression read-out (evident in the variability in expression values between different probes for the same gene). However, there is some correlation between gene expression and protein expression and it is likely that high gene expression will correlate with protein expression.

To identify new cMS mutations, PCR primers were designed to amplify cMS-containing regions of the selected genes (Supplementary Data 1). PCR and DNA sequencing was performed using protocols described above. Five MSI-High colon cancer cell lines (HCT116, LoVo, LIM1215, LS174T and HCA7) and one MSS cell line (SW480) were initially screened for frameshift mutations in the selected genes. Genes bearing mutations in this initial screen were also tested for mutations in the remaining 6 MSI colon cancer cell lines.

### *In silico* T cell epitope prediction

Mutant proteins predicted to be generated from common cMS mutations were screened for likely HLA-A*0201 CTL epitopes, using the publicly available predictive software, SYFPEITHI[26]. This program uses a scoring system based upon the binding motifs of known CTL epitopes to generate binding scores that rank all potential nonamer and decamer peptides generated from the input protein sequences in order of likely binding to the HLA-A*0201 molecule. Peptides with scores in the top 2% of peptides were identified as likely CTL epitopes, the scores of which were validated by comparison with score of 133 known HLA-A*0201 CTL epitopes listed on the SYFPEITHI website[26] and 2 MSI-derived HLA-A*0201 epitopes[13], [14] not listed at this site.

## Supporting Information

Figure S1 **Proteomic analysis for CREBBP and EP300.** (A) Immunoprecipitates of SW480 (wild type CREBBP) and LoVo (−1, wt) cell lines were prepared using anti‐CREBBP antibody and (B) immunoprecipitates of LIM1215 (wild type EP300) and HCT116 (−1, wt) cell lines were prepared using anti‐EP300 antibody. Tryptic digests were prepared from the region of the gel including the suspected CREBBP and EP300 bands. Tandem mass spectrometry revealed multiple peptide sequences corresponding to CREBBP in the SW480 cell line and EP300 in the LIM1215 cell line, confirming the specificity of the wild type protein band. Mutant protein bands were not confirmed by this technique, possibly due to the presence of more abundant co‐immunoprecipitated proteins such as MYH9 (data not shown).
(DOCX)

Figure S2 **Gene expression to prioritise target gene selection.** NMD‐R transcripts with cMS ≥8 mer and mutant C‐terminus ≥30 a.a. and CREBBP were assessed using colorectal cancer gene expression data from two studies. Boxplots of top 50 gene expression levels shown, with interquartile range above non‐specific background (set at 6). High gene expression was used to indicate likely protein expression.
(DOC)

Table S1 **Coding microsatellite (cMS) sequencing data for genes with previously reported cMS mutations.**
(XLS)

Table S2 a. Genes with mononucleotide cMS predicted to generate NMD‐resistant transcripts (−1 base cMS frameshift mutation). b. Genes with mononucleotide cMS predicted to generate NMD‐resistant transcripts (−2 base cMS frameshift mutation). c. Genes with dinucleotide cMS predicted to generate NMD‐resistant transcripts (−1 repeat cMS fraemshift mutation). d. Genes with dinucleotide cMS predicted to generate NMD‐resistant transcripts (−2 repeat cMS frameshift mutation).
(XLS)

Table S3 **Genes with at least 8 mer cMS predicted to generate NMD‐resistant transcripts with mutant C‐terminus at least 10 amino acids in length.**
(XLSX)

Table S4 **Coding microsatellite (cMS) sequencing data for genes selected from genome‐wide search for NMD‐resistant transcripts.**
(XLS)

Table S5 NMD‐R transcripts assessed for potential HLA‐A*0201 epitopes.
(XLSX)

Table S6 Syfpeithi binding scores for known HLA‐A*0201 ligands.
(XLSX)

Table S7 **PCR primer details.**
(XLSX)
