# Correction: Nonsense Mediated Decay Resistant Mutations Are a Source of Expressed Mutant Proteins in Colon Cancer Cell Lines with Microsatellite Instability

**DOI:** 10.1371/annotation/53805ecf-7d10-4d99-9cec-f27f5e0d4166

**Published:** 2011-01-13

**Authors:** David S. Williams, Matthew J. Bird, Robert N. Jorissen, Yen Lin Yu, Francesca Walker, Hui Hua Zhang, Edouard C. Nice, Antony W. Burgess

The fifth author's name was spelled incorrectly. The correct name is: Francesca Walker. 

