# Supplementary material for: Nonsense Mediated Decay Resistant Mutations Are a Source of Expressed Mutant Proteins in Colon Cancer Cell Lines with Microsatellite Instability
Source: PLoS One. 2010 Dec 31;5(12):e16012. doi: 10.1371/journal.pone.0016012 (PMC3013145; doi:10.1371/journal.pone.0016012)
Supplement: Figure S1 — Proteomic analysis for CREBBP and EP300. (A) Immunoprecipitates of SW480 (wild type CREBBP) and LoVo (−1, wt) cell lines were prepared using anti‐CREBBP antibody and (B) immunoprecipitates of LIM1215 (wild type EP300) and HCT116 (−1, wt) cell lines were prepared using anti‐EP300 antibody. Tryptic digests were prepared from the region of the gel including the suspected CREBBP and EP300 bands. Tandem mass spectrometry revealed multiple peptide sequences corresponding to CREBBP in the SW480 cell line and EP300 in the LIM1215 cell line, confirming the specificity of the wild type protein band. Mutant protein bands were not confirmed by this technique, possibly due to the presence of more abundant co‐immunoprecipitated proteins such as MYH9 (data not shown). (DOCX) [file pone.0016012.s001.docx]

**Figure S1. Proteomic analysis for CREBBP and EP300.**

(A) Immunoprecipitates of SW480 (wild type CREBBP) and LoVo (-1, wt) cell lines were prepared using anti-CREBBP antibody and (B) immunoprecipitates of LIM1215 (wild type EP300) and HCT116 (-1, wt) cell lines were prepared using anti-EP300 antibody. Tryptic digests were prepared from the region of the gel including the suspected CREBBP and EP300 bands. Tandem mass spectrometry revealed multiple peptide sequences corresponding to CREBBP in the SW480 cell line and EP300 in the LIM1215 cell line, confirming the specificity of the wild type protein band. The mutant protein bands were not confirmed by this technique, possibly due to the presence of more abundant co-immunoprecipitated proteins such as MYH9 (data not shown).
