# Supplementary material for: Nonsense Mediated Decay Resistant Mutations Are a Source of Expressed Mutant Proteins in Colon Cancer Cell Lines with Microsatellite Instability
Source: PLoS One. 2010 Dec 31;5(12):e16012. doi: 10.1371/journal.pone.0016012 (PMC3013145; doi:10.1371/journal.pone.0016012)
Supplement: Figure S2 — Gene expression to prioritise target gene selection. NMD‐R transcripts with cMS ≥8 mer and mutant C‐terminus ≥30 a.a. and CREBBP were assessed using colorectal cancer gene expression data from two studies. Boxplots of top 50 gene expression levels shown, with interquartile range above non‐specific background (set at 6). High gene expression was used to indicate likely protein expression. (DOC) [file pone.0016012.s002.doc]

**Figure S2. Gene expression to prioritise target gene selection.** NMD-R transcripts with cMS ≥ 8mer and mutant C-terminus ≥ 30 a.a. and CREBBP were assessed using colorectal cancer gene expression data from two studies. Boxplots of top 50 gene expression levels shown, with interquartile range above non-specific background (set at 6). High gene expression was used to indicate likely protein expression.

Top expression values (Andersen set)

Top expression values (Watanabe set)
